# Influence of reconstruction kernels on the accuracy of CT-derived fractional flow reserve

**DOI:** 10.1007/s00330-021-08348-0

**Published:** 2021-11-04

**Authors:** Fabian Ammon, Maximilian Moshage, Silvia Smolka, Markus Goeller, Daniel O. Bittner, Stephan Achenbach, Mohamed Marwan

**Affiliations:** Department of Cardiology, Friedrich-Alexander University Erlangen-Nürnberg (FAU), Ulmenweg 18, 91054 Erlangen, Germany

**Keywords:** Computed tomography angiography, Image processing, computer-assisted, Fractional flow reserve, myocardial, Coronary artery disease

## Abstract

**Objectives:**

We evaluated the influence of image reconstruction kernels on the diagnostic accuracy of CT-derived fractional flow reserve (FFR_CT_) compared to invasive FFR in patients with coronary artery disease.

**Methods:**

Sixty-nine patients, in whom coronary CT angiography was performed and who were further referred for invasive coronary angiography with FFR measurement via pressure wire, were retrospectively included. CT data sets were acquired using a third-generation dual-source CT system and rendered with medium smooth (Bv40) and sharp (Bv49) reconstruction kernels. FFR_CT_ was calculated on-site using prototype software. Coronary stenoses with invasive FFR ≤ 0.80 were classified as significant. Agreement between FFR_CT_ and invasive FFR was determined for both reconstruction kernels.

**Results:**

One hundred analyzed vessels in 69 patients were included. Twenty-five vessels were significantly stenosed according to invasive FFR. Using a sharp reconstruction kernel for FFR_CT_ resulted in a significantly higher correlation with invasive FFR (*r* = 0.74, *p* < 0.01 vs. *r* = 0.58, *p* < 0.01; *p* = 0.04) and a higher AUC in ROC curve analysis to correctly identify/exclude significant stenosis (AUC = 0.92 vs. AUC = 0.82 for sharp vs. medium smooth kernel, respectively, *p* = 0.02). A FFR_CT_ value of ≤ 0.8 using a sharp reconstruction kernel showed a sensitivity of 88% and a specificity of 92% for detecting ischemia-causing lesions, resulting in a diagnostic accuracy of 91%. The medium smooth reconstruction kernel performed worse (sensitivity 60%, specificity 89%, accuracy 82%).

**Conclusion:**

Compared to invasively measured FFR, FFR_CT_ using a sharp image reconstruction kernel shows higher diagnostic accuracy for detecting lesions causing ischemia, potentially altering decision-making in a clinical setting.

**Key Points:**

*• Image reconstruction parameters influence the diagnostic accuracy of simulated fractional flow reserve derived from coronary computed tomography angiography.*

*• Using a sharp kernel image reconstruction algorithm delivers higher diagnostic accuracy compared to medium smooth kernel image reconstruction (gold standard invasive fractional flow reserve).*

## Introduction

Coronary computed tomography (CT) is an established anatomic imaging modality for exclusion and detection of coronary artery stenoses; it carries a “class I” indication for the workup of coronary artery disease (CAD) in specific patient populations. However, assessment of the physiologic relevance of coronary stenoses based on anatomy remains difficult and ischemia testing for guiding revascularization decisions is recommended.

In the absence of sufficient information from previous stress testing, the hemodynamic significance of coronary stenoses can be assessed with invasive fractional flow reserve (FFR). An FFR-based approach has repeatedly been shown to be superior to relying on angiography alone regarding clinical outcome [1–4], and FFR-based decision-making has emerged as the gold standard for performing or deferring percutaneous coronary intervention [5].

With the help of computational fluid dynamics, non-invasive FFR values are generated from coronary computed tomography angiography (cCTA) data sets (CT-derived FFR, FFR_CT_) and thus add hemodynamic information to pure anatomic images [6].

Several trials have reported a good correlation of FFR_CT_ with invasive FFR. Furthermore, compared to invasive FFR, the diagnostic accuracy of FFR_CT_ for detecting ischemia-causing coronary lesions could be shown to be better than cCTA alone [7–9]. Minimizing artifacts and the use of nitroglycerin and beta blocker are key factors for achieving a high diagnostic accuracy of FFR_CT_ [7–9].

Since the determination of FFR_CT_ requires a high degree of anatomic information and spatial resolution, it is conceivable that the choice of reconstruction kernels used in the process of generating CT image data sets impacts the results of FFR_CT_. Reconstruction kernels can be compared to image filters that determine the balance between image noise and spatial resolution within the obtained data set. A “sharp” reconstruction kernel is very useful for assessment of coronary arteries when pronounced atherosclerosis is present and especially in the presence of calcifications. The influence of image reconstruction kernels on the diagnostic performance of FFR_CT_ has not yet been investigated.

The aim of this study was to assess the influence of using “sharp” versus “smooth” reconstruction kernels on the accuracy of FFR_CT_, which was determined by comparison to invasively measured FFR.

## Materials and methods

### Study design and patient population

This is a single-center, retrospective analysis. Patients referred for cCTA between April 2015 and February 2019 were screened for inclusion in this analysis. Exclusion criteria were non-diagnostic cCTA data sets, coronary anomalies, the presence of a chronic total obstruction or prior stent implantation in the vessel of interest, and any status post coronary artery bypass grafting as well as the presence of ostial left main or ostial right coronary stenosis. Sixty-nine patients with an invasive coronary angiogram within 2 months after cCTA and invasive FFR measurement during which the wire position was unambiguously documented by fluoroscopy were included in this study.

### CT data acquisition and image reconstruction

Coronary CT angiography was performed using a third-generation dual-source CT system (Somatom Force, Siemens Healthineers). Patients with heart rates > 60 bpm were given atenolol p.o. and/or metoprolol i.v. All patients received nitroglycerin sublingually prior to the scan. The acquisition protocol was chosen according to patient characteristics. ECG-triggered prospective axial acquisition was performed in most patients. Patients with persistent heart rates > 70 bpm in spite of medication or with frequent ectopic beats were examined using spiral acquisition with retrospectively ECG-gated image reconstruction. Patients < 45 years old with heart rates < 55 bpm were examined using prospectively ECG-triggered high-pitch spiral acquisition. Coronary calcium screening CT was performed in most patients before administering contrast agent. The scan range extended from the proximal ascending aorta to the caudal aspect of the heart. For coronary CT angiography, tube voltage (ranging from 90 to 120 mV) and tube current time product (450 to 650 mAs) were adjusted to the patient’s body weight and calcium score. Contrast agent transit time was determined by giving a bolus of 10 ml of contrast agent (Ultravist 370®, Bayer vital GmbH GB Pharma). For CT angiography, 50 ml of contrast agent at a flow rate of 5 ml/s, followed by a 50-ml saline chaser (20% contrast agent) at the same flow rate, was injected.

**Fig. 1:**
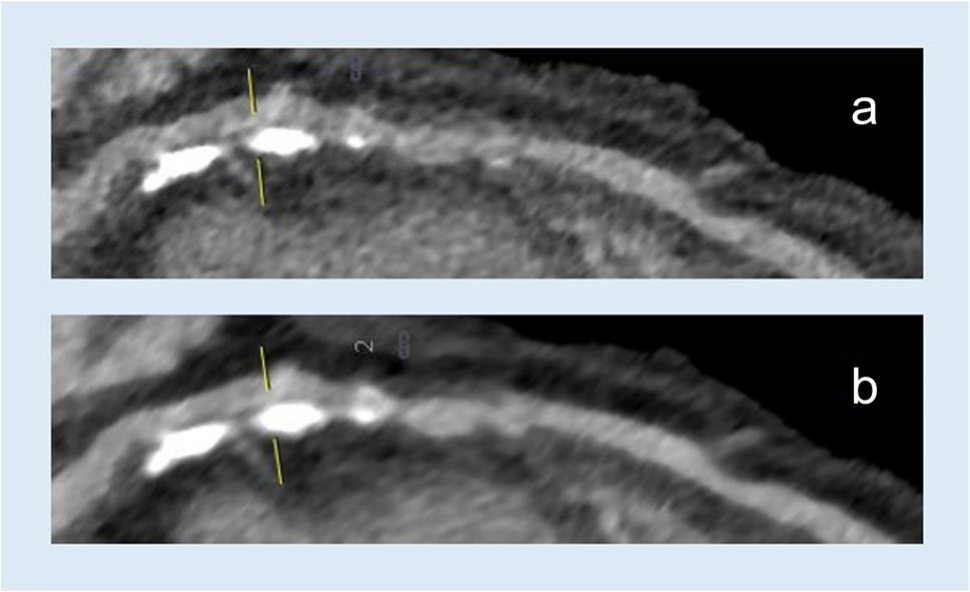
cCTA image with a longitudinal section of the left anterior descending artery with a (**a**) sharp Bv49 kernel and (**b**) medium smooth Bv40 kernel

All image data sets were reconstructed with a slice thickness of 0.5 mm, increment of 0.3 mm, and a matrix of 512. Iterative reconstruction (Admire®, Siemens Healthineers) at a strength level of 2 was used for all data sets. Reconstructions were rendered using both a medium soft convolution kernel (Siemens Bv40) and a sharp convolution kernel (Siemens Bv49), with otherwise unchanged parameters (Fig. 1).

### *Calculation of FFR*_*CT*_

**Fig. 2:**
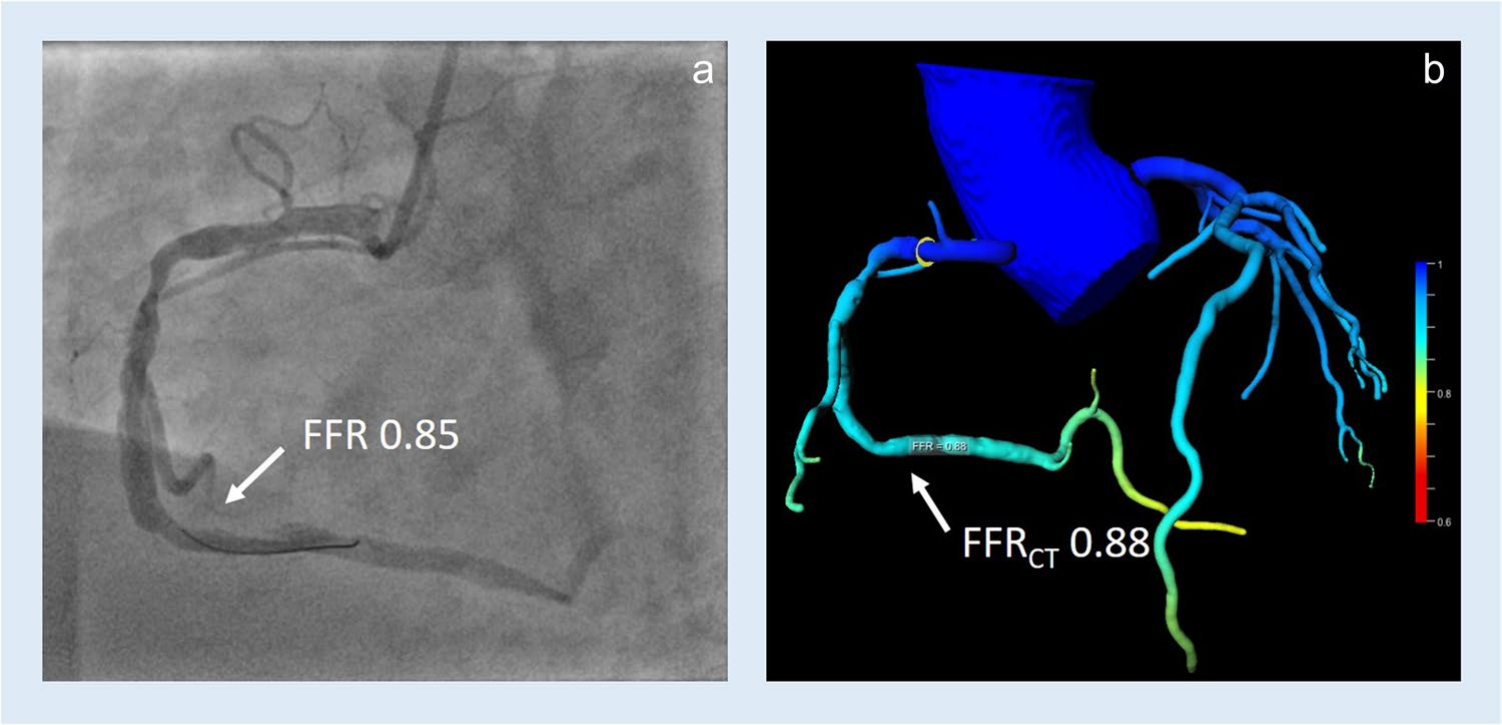
**a** Invasive angiogram with an FFR wire placed in the distal third of the right coronary artery. **b** 3D CT reconstruction of the coronary tree showing FFR_CT_

**Fig. 3:**
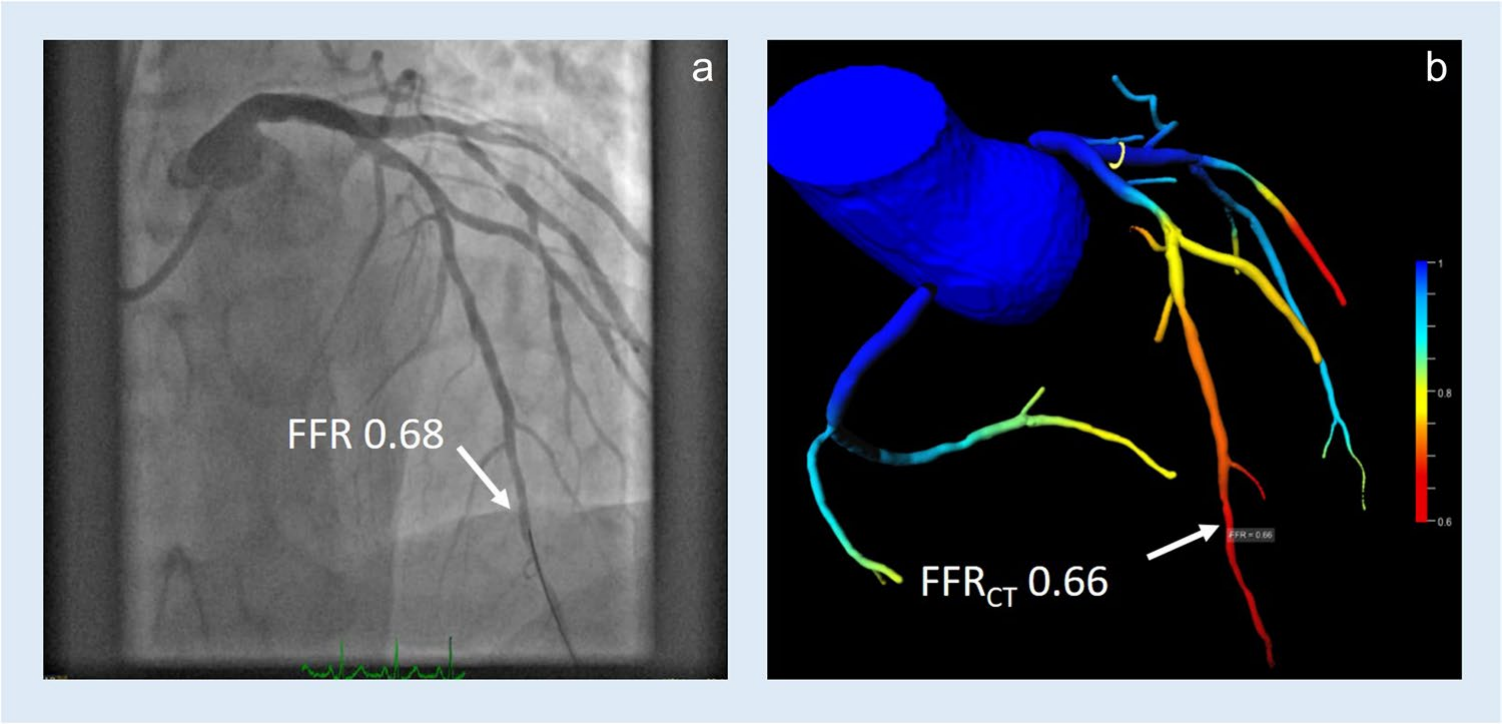
**a** Invasive angiogram with an FFR wire placed in the distal left anterior descending artery. **b** 3D CT reconstruction of the coronary tree showing FFR_CT_

FFR_CT_ was calculated on-site using a PC-based prototype software (cFFR version 3.0, Siemens Healthineers). The program automatically renders the centerline and lumen of the vessels. Manual correction by a user blinded to invasive FFR was done where necessary. When completed, a 3D model of the coronary tree is generated with FFR values at any point on the vessel. After identifying the exact location of the FFR wire in the invasive coronary angiogram, the FFR_CT_ value at the identical location of the simulated coronary tree was noted and compared to the invasive FFR value (Figs. 2 and 3).

### Invasive FFR measurement

Invasive FFR was measured using PressureWire X Guidewire (St. Jude Medical/Abbott) after producing hyperemia with adenosine intracoronary. Coronary stenoses with invasively measured FFR ≤ 0.80 were classified as hemodynamically significant. Agreement between FFR_CT_ using both reconstruction parameters was compared to invasive FFR.

### Statistical analyses

All statistical analyses were done with IBM® SPSS® Statistics (version 24). Continuous variables are presented as mean ± standard deviation or median (interquartile range [IQR]). Wilcoxon test was used to compare groups. Results were deemed significant with a *p* value < 0.05. Receiver operating characteristic (ROC) curve analyses were performed and ROC curves and correlations compared with MedCalc Software Ltd.

## Results

Out of 72 patients screened for inclusion in this analysis, 3 patients had to be excluded because of insufficient image quality of cCTA for the calculation of FFR_CT_ and one patient due to technical reasons with FFR_CT_ measurement. Sixty-nine patients (mean age 63 ± 10 years, 18 women) were included in this study. Baseline clinical characteristics are shown in Table 1.

**Table 1:** Demographic and clinical characteristics of included patients

	All patients (*N* = 69)
Mean age—yr	63 ± 10
Known coronary artery disease* (CAD)—no. (%)	3 (4)
Arterial hypertension—no. (%)	55 (80)
Diabetes mellitus—no. (%)	11 (16)
Hyperlipoproteinemia—no. (%)	49 (71)
Positive family history for CAD—no. (%)	21 (30)
Prior or current smoker—no. (%)	28 (41)
Body mass index—kg/m^2^	27.3 ± 3.9
Mean echocardiographic ejection fraction—%	56.6 ± 7.0
Angiographic degree of stenosis—%	57.2 ± 15.8
Pure diagnostic coronary angiography—no. (%)	33 (48)

^*^Previous percutaneous coronary intervention not in the analyzed vessel

Invasive coronary angiography was performed after a mean time of 8 days after cCTA. A total of 100 vessels were investigated both by invasive and CT-based FFR. The invasive angiographic lumen reduction of the target vessel ranged between 30 and 90% on visual analysis (mean 57%). Twenty-seven vessels had a degree of stenosis of 30– < 50%, 44 vessels showed a stenosis of 50– < 70%, while in 29 vessels a lumen reduction of 70–90% was found. In case of multiple stenoses, the pressure wire was placed after the most distal stenosis. The median invasive FFR value was 0.87 (IQR 0.14), and a total of 25 analyzed vessels (25% of all vessels) were classified as hemodynamically significantly stenosed due to an invasive FFR value of ≤ 0.8.

In most cases (57/100), the left anterior descending artery (LAD) with its side branches was the vessel of interest, followed by the circumflex coronary artery (CX, 27/100) and the right coronary artery (RCA, 16/100). The hemodynamic relevance of lesions according to invasive FFR was detected in the LAD territory in 20 cases (35% of all measured LAD), in the CX territory in 3 cases (11% of all measured CX), and in the RCA in 2 cases (13% of all measured RCA). There was a significant association between the vessel of interest and the severity of the stenosis (*p* = 0.027).

The median FFR_CT_ using a medium smooth reconstruction kernel (Bv40) was 0.87 (IQR 0.11), and the median FFR_CT_ using a sharp reconstruction kernel (Bv49) was 0.87 (IRQ 0.13). While FFR_CT_ values simulated with Bv40 were not relevantly different compared with invasive FFR values (*p* = 0.26), this was the case when simulated with Bv49 (*p* = 0.017).

**Fig. 4:**
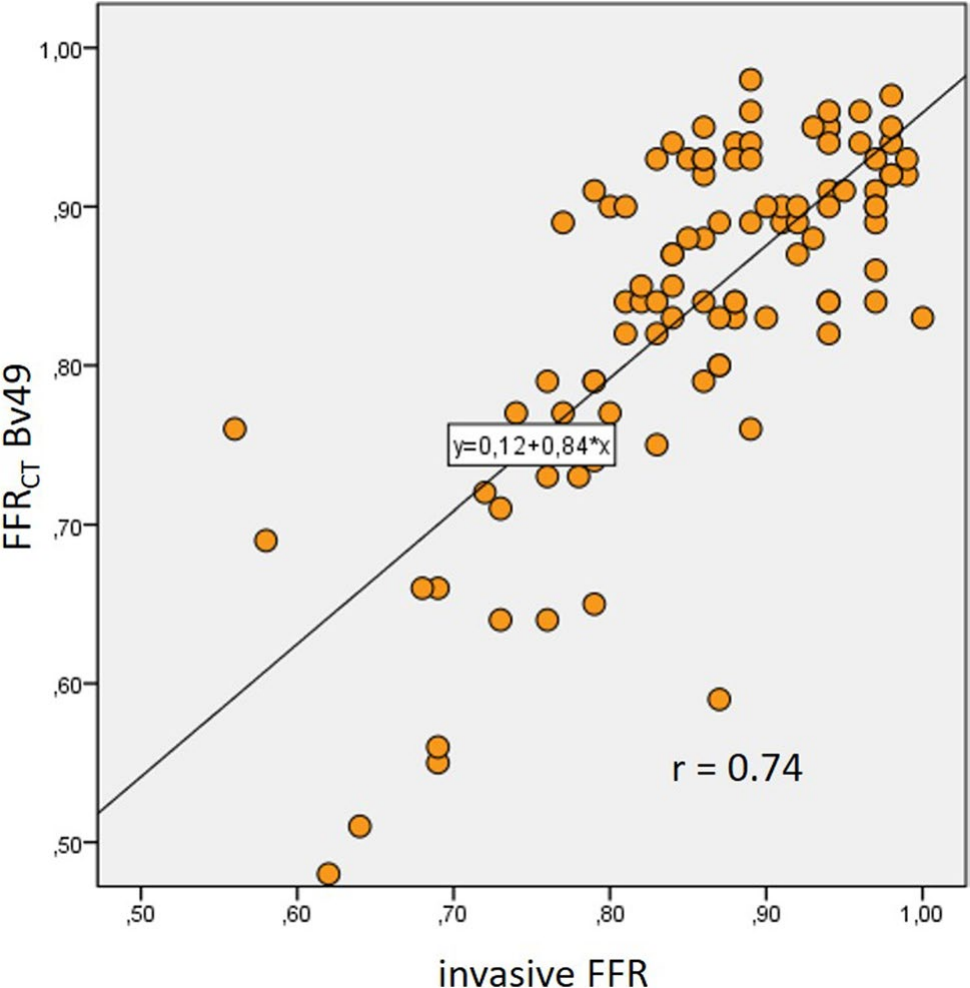
Scatterplot with FFR_CT_ Bv49 and invasive FFR

**Fig. 5:**
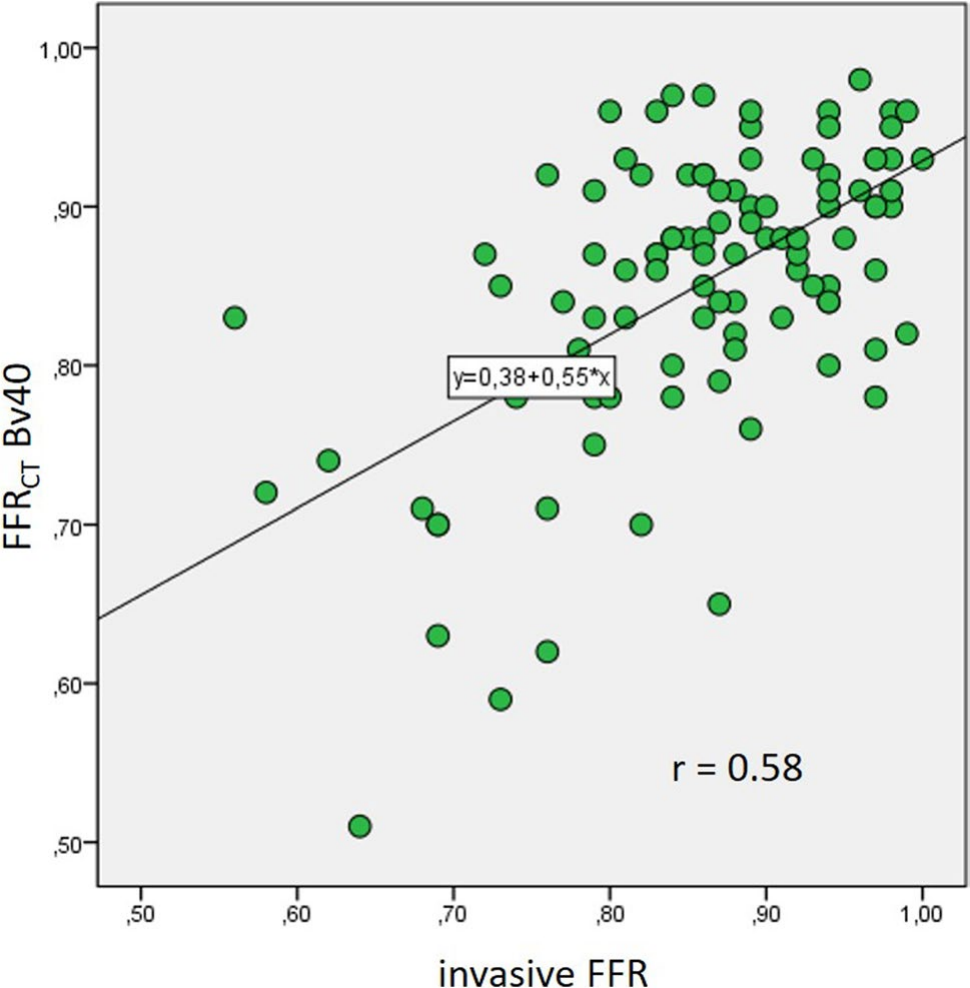
Scatterplot with FFR_CT_ Bv40 and invasive FFR

Using the sharp reconstruction kernel, a significantly closer correlation of FFR_CT_ with invasive FFR was observed in comparison to medium smooth reconstruction kernel (*r* = 0.74, *p* < 0.0001 vs *r* = 0.58, *p* < 0.0001; direct comparison of correlation coefficients *p* = 0.04; scatterplots Figs. 4 and 5). ROC curve analysis showed an area under the ROC curve (AUC) of 0.92 (*p* < 0.001) for FFR_CT_ using a sharp reconstruction kernel to correctly identify or exclude hemodynamically significant stenosis compared to invasive FFR, whereas a smaller AUC of 0.82 (*p* < 0.001) was observed for a medium smooth reconstruction kernel. This difference of AUC between Bv49 and Bv40 FFR_CT_ was statistically significant (*p* = 0.02). A FFR_CT_ value of ≤ 0.8 using sharp reconstruction kernel data sets showed a sensitivity of 88% and a specificity of 92% to detect diseased vessels causing ischemia compared to the invasive gold standard, resulting in an accuracy of 91%. The medium smooth kernel data sets performed significantly worse, having a sensitivity of 60% and specificity of 89%, resulting in an accuracy of 82% when using the same FFR_CT_ cut-off value of ≤ 0.8. Statistical analysis [10] demonstrated the superiority of sharp kernel reconstructions regarding diagnostic accuracy when compared to medium smooth kernel reconstructions (*p* = 0.02).

In 46 patients, only one vessel was assessed for FFR whereas in 23 patients, more than one vessel was assessed. On a per-patient basis with one pathologic invasive FFR value indicating the presence of relevant stenoses, FFR_CT_ with a Bv49 reconstruction kernel was able to correctly classify 61 patients. In 5 patients, the severity of stenosis was overestimated, and in 3 patients, relevant stenoses were not detected (sensitivity 88%, specificity 89%, diagnostic accuracy 88%). Using a Bv40 reconstruction kernel, only 57 patients could be correctly classified with 5 patients overestimating and 7 patients underestimating the degree of stenosis severity (sensitivity 71%, specificity 89%, diagnostic accuracy 83%). The numerically higher diagnostic accuracy of a sharp reconstruction kernel was not significantly superior to a medium smooth reconstruction kernel on a per-patient basis (*p* = 0.14).

## Discussion

To the best of our knowledge, this is the first study to evaluate the influence of the image reconstruction kernel on FFR_CT_ values. We could show that FFR_CT_ using a sharper reconstruction kernel had a significantly better correlation with invasive FFR compared to a medium smooth reconstruction kernel. These results are of clinical relevance as patient-related decision-making concerning further diagnostic workup or invasive assessment critically depends on the accuracy of the FFR_CT_ values. The higher spatial resolution of CT data sets generated by a sharper reconstruction kernel outweighs the disadvantage of increased noise, and therefore, the preferred kernel should render high-spatial-resolution data sets (often termed “sharp”). High image quality and high spatial resolution are key factors for obtaining accurate FFR_CT_ results since the hemodynamic significance of a lesion is partly affected by the depicted degree of stenosis length and vessel diameter. Presumably, the enhanced spatial resolution offered by sharper reconstruction kernels improves automatic delineation of coronary centerlines with better lumen depiction and subsequent manual corrections can be done more confidently when needed.

Baumann and Renker et al compared invasive FFR with FFR_CT_ by a prior version of the software used in our study. They showed a correlation similar to ours between FFR_CT_ and invasively measured FFR (*r* = 0.74, *p* < 0.0001) in a cohort of 28 patients/36 vessels [11]. In another trial with 53 patients and 67 lesions, the results with on-site FFR_CT_ calculation were similar: a reasonable correlation of FFR_CT_ with invasive FFR (*r* = 0.66, *p* < 0.001) and increased diagnostic performance of FFR_CT_ over cCTA alone for the detection of hemodynamically relevant stenosis defined by an invasive FFR < 0.8 [12]. The image reconstruction kernel was not specified in the first trial, but in the second publication, image reconstruction was performed using a vascular kernel, B26f, which corresponds to a medium smooth reconstruction kernel. A study with 71 patients/91 vessels performed in our department could demonstrate a higher correlation (*r* = 0.85) and high diagnostic accuracy for FFR_CT_ compared to invasive FFR by using the sharp Bv49 kernel [13].

The mean diameter reduction in the target lesion was 57% in our study. Invasive FFR is especially suitable to evaluate lesions with intermediate stenosis, as stenosis as low as 30–50% can already have hemodynamic relevance [1] [14], while no additional benefit has been shown for lesions showing > 90% stenosis [15]. Thus, our study investigated the effect of image reconstruction kernels in a study population, which represents the daily life challenge of evaluating the usefulness of performing a percutaneous coronary intervention.

Coronary CT angiography is recommended by the guidelines of the European Society of Cardiology to clarify whether CAD causes chest pain when clinical assessment alone cannot exclude CAD [16]. However, the low specificity of cCTA to rule out hemodynamic CAD sets patients at risk of unnecessary non-invasive testing or invasive coronary angiography [17, 18]. The initial SCOT-HEART trial roused suspicion of increased invasive testing of patients presenting with chest pain who received coronary CT angiography as a first-line diagnostic tool for evaluation of chest pain [19]. However, the 5-year results of the SCOT-HEART trial could not only show a decrease in the primary endpoint, but also that the higher rate of invasive coronary angiography could only be observed in the first few months, while later no difference could be found [20]. Potentially using non-invasive FFR measurement for evaluating coronary stenoses in coronary CT angiography could have helped to reduce the number of early coronary angiographies while maintaining the positive long-term effect of increased use of antithrombotic and cholesterol-lowering agents in this cohort. Curzen et al could show that the availability of FFR_CT_ values changes the treatment plan in about one-third of patients when added to standard coronary CT angiography [21]. The PLATFORM study also demonstrated that off-site FFR_CT_ can safely reduce the number of invasive coronary angiographies without prognostic disadvantages in the group of patients with canceled invasive diagnostic in 1 year follow-up [22].

We could show that FFR_CT_ performed with data reconstructed with a sharp kernel shows better results when compared to invasive FFR than a medium smooth kernel. The use of a sharp reconstruction kernel thus can help to achieve better diagnostic accuracy of non-invasive FFR_CT_ algorithms.

Several limitations need to be acknowledged in this study. The number of patients and FFR-measured vessels are relatively small. Particularly, the number of pathologic invasive FFR values was low with 25 vessels. However, this is no sign of the low specificity of cCTA, as severely stenosed vessels were frequently not FFR-measured but directly intervened and only the intermediate stenoses were evaluated by FFR. With a mean angiographic degree of stenosis of 57%, it was to be expected that the number of pathologic FFR is rather low, owing that even in vessels with 50 to 70% stenosis, hemodynamic significance of the lesion is present only in about one-third of cases [15]. The predominance of left anterior descending artery lesions is reflecting the wider use of FFR in this vessel as a larger retrospective trial has already observed [23]. An inherent referral bias cannot be excluded as only patients with lesions deemed to be significant in cCTA were further sent for clinical invasive clarification. FFR_CT_ measurements for both kernels were done by the same operator, so bias cannot be excluded totally, although the operator was strictly blinded for the invasive FFR value. Moreover, we analyzed the influence of the reconstruction kernel on FFR_CT_ values within the same data set. The potential influence of other acquisition parameters on CT-derived FFR including tube current and output, and collimation and contrast opacification of the coronary arteries, was not analyzed in this cohort. Furthermore, this is a single-vendor study, and further research is needed to generalize the results to other software simulating FFR_CT_.

In conclusion, we could demonstrate that image reconstruction parameters have a significant impact on the diagnostic performance of FFR_CT_. A sharper reconstruction kernel provides a higher correlation with invasive FFR, potentially changing clinical decision-making.

## Abbreviations

AUC: Area under the ROC curve
bpm: Beats per minute
CAD: Coronary artery disease
cCTA: Coronary computed tomography angiography
CT: Computed tomography
CX: Circumflex coronary artery
FFR: Fractional flow reserve
FFR_CT_: CT-derived FFR
i.v.: Intravenous
IQR: Interquartile range
LAD: Left anterior descending artery
p.o.: Per os
RCA: Right coronary artery
ROC: Receiver operating characteristic

## Declarations

### Guarantor

The scientific guarantor of this publication is Fabian Ammon.

### Statistics and biometry

No complex statistical methods were necessary for this paper.

### Methodology

• retrospective

• diagnostic study

• performed at one institution
